# Clinical and virological effects of the integrase inhibitor raltegravir in cats with naturally progressive feline leukaemia virus infection

**DOI:** 10.1177/1098612X261452556

**Published:** 2026-05-13

**Authors:** Pedro Morais de Almeida, Adriana Belas, João Martins, Rita Picado, André Meneses, Joana Tavares de Oliveira, Carlos Viegas

**Affiliations:** 1 Faculty of Veterinary Medicine, Lusófona University-Lisbon University Centre, Portugal; 2 Animal and Veterinary Research Center (CECAV), University of Trás-os-Montes and Alto Douro, Vila Real, Portugal; 3 Polytechnic Institute of Lusofonia (IPLUSO), School of Health, Protection and Animal Welfare, Lisbon, Portugal; 4 I-MVET- Research in Veterinary Medicine, Faculty of Veterinary Medicine, Lusófona University-Lisbon University Centre, Portugal; 5 Animal and Veterinary Research Center (CECAV), Lusófona University-Lisbon University Centre, Portugal; 6 Department of Veterinary Sciences, School of Agricultural and Veterinary Sciences (ECAV), University of Trás-os-Montes and Alto Douro, Vila Real, Portugal; 7 CIVG-Vasco da Gama Research Center, University School Vasco da Gama, Lordemão, Portugal

**Keywords:** Antiviral, FeLV-related conditions, survival time, proviral DNA load, viral RNA load

## Abstract

**Objectives:**

Feline leukaemia virus (FeLV) infection – particularly the progressive course – continues to cause substantial morbidity and reduced survival in cats. Therapeutic options with proven antiviral effectiveness remain limited. This study aimed to evaluate the clinical and virological effects of the integrase inhibitor raltegravir in naturally infected, FeLV-progressive cats with FeLV-related conditions, clinical outcome, viraemia, proviral burden and survival.

**Methods:**

In total, 14 client-owned cats with confirmed progressive FeLV infection and at least one FeLV-related condition were enrolled. Raltegravir was administered for 90 days, followed by a 90-day treatment-free observation period. Clinical evaluation and quantification of viral RNA and proviral DNA loads were performed at treatment initiation, day 45, day 90 and day 180. Cats continued to receive standard of care as clinically indicated. Survival data were recorded until study closure.

**Results:**

After 45 days of treatment, plasma viral RNA load showed a non-significant mean reduction of 1.34 log_10_ (*P* = 0.204). At day 90, mean viral load continued to decrease, with reductions of 1.10 log_10_ at 40 mg (*P* = 0.208) and 1.39 log_10_ at 80 mg (*P* = 0.195), none of which reached statistical significance. Raltegravir did not exert a consistent effect on proviral DNA load. Most FeLV-related conditions remained clinically stable or improved during the 180-day monitoring period. Leukaemia and lymphoma were the main causes of death. Median survival time was 48 months from FeLV diagnosis and 10.8 months from treatment initiation.

**Conclusions and relevance:**

To the authors’ knowledge, this is the first prospective longitudinal study in naturally infected, FeLV-progressive cats with FeLV-related conditions assessing the effects of raltegravir on viraemia, proviral load and clinical outcomes. These real-world data suggest that raltegravir may be associated with numerical reductions in viraemia and clinical stabilisation in some cats. A definitive virological or survival benefit, however, could not be demonstrated. Longer term controlled studies – potentially within multimodal antiviral strategies – are warranted to further define its therapeutic role.

## Introduction

Feline leukaemia virus (FeLV) remains common in several regions, particularly southern Europe, South America and Africa.^1,2^ Adult intact male cats with outdoor access and aggressive behaviour remain at increased risk.^3–7^

FeLV causes a broad spectrum of severe disease, including haematological disorders, neoplasia, immune-mediated conditions, immunosuppression and bone marrow dysfunction, and remains one of the feline viral infections associated with the highest morbidity and a marked reduction in survival, particularly in progressively infected cats.^8–12^ Accurate diagnosis is therefore crucial, as the four recognised infection courses differ markedly in clinical presentation and survival.^13–17^ However, distinguishing between them is difficult owing to the complex, dynamic interaction between viral factors and host immunity, which determines proviral integration and the ultimate clinical course.^4,18,19^

Management of progressively infected cats has relied largely on immunomodulatory approaches, such as recombinant human interferon-alfa (IFN-α)^20–22^ and feline interferon-omega (IFN-ω).^23^ One study reported transient reductions in viral parameters after treatment with IFN-α,^22^ although these effects were not sustained. These agents can improve clinical status and survival – mainly by enhancing resistance to secondary infections^21,24^ – but controlled studies have not demonstrated consistent virological benefit.^23,25,26^

Novel strategies, including synthetic peptides^27^ and gene therapy,^28^ have so far been explored only sporadically in vitro. More recently, CRISPR/SaCas9-assisted gene therapy has shown in vitro promise in lowering viral loads in progressively infected cats, potentially enabling immune-mediated control and a shift towards regressive infection.^29^ In contrast to immunotherapy^30,31^ and bone marrow transplantation,^32^ which remain largely experimental even in human medicine, antiviral therapy currently offers the most pragmatic antiviral option.^17,26,33,34^

Only a few antiviral agents used in clinical practice have shown in vivo efficacy with acceptable safety in FeLV, particularly in naturally infected cats, targeting different stages of the viral replication cycle, most notably the nucleoside analogue reverse transcriptase inhibitor zidovudine (AZT), administered off-label using human formulations.^17,26,35,36^ This antiviral inhibits reverse transcription but shows limited benefit in established infection^20,37^ and can cause clinically significant adverse effects, including non-regenerative anaemia, being contraindicated at higher doses in cats with erythroid leukaemia.^38,39^

Raltegravir, an integrase inhibitor, reduces FeLV replication in vitro.^40,41^ Although mainly metabolised by glucuronidation, which is limited in cats,^42,43^ raltegravir appears safe and well tolerated in this species.^44^ In an experimental model of progressive FeLV infection (seven cats), viral RNA load began to fall after 5 weeks of treatment, reaching an approximately five-fold reduction at 9 weeks, but rebounded in all cats after drug discontinuation.^44^

Over a decade later, only two studies have assessed raltegravir in naturally infected cats: two case reports of regressive FeLV-infected cats^45^ and a case series of 10 progressively infected cats, some receiving additional antivirals, with limited published clinical characterisation and virological reporting, including assessment of viral RNA load and p27 antigen levels at follow-up intervals.^46^ Despite this limited evidence, raltegravir use in clinical practice is increasing, often in combination protocols for FeLV-associated disease.^47–50^ Consequently, data on the clinical and virological effects of antiviral therapy in naturally FeLV-progressive cats, particularly regarding long-term outcomes and survival, remain scarce.

This prospective longitudinal single-arm interventional study aimed to assess the effect of short-term treatment with the integrase inhibitor raltegravir on the clinical evolution of FeLV-related conditions, median survival time (MST) and viraemia in naturally FeLV-progressive cats. Changes in clinical parameters, life expectancy, proviral DNA load and viral RNA load were evaluated. We hypothesised that raltegravir would reduce viral RNA load and improve clinical condition, thereby increasing life expectancy compared with that typically reported for this infection course.

## Materials and methods

### Eligibility criteria and case definition

This was a prospective, longitudinal, single-arm interventional study (January 2022–September 2025) assessing clinical and virological responses to raltegravir in 14 naturally infected, progressively FeLV-positive cats with FeLV-related disease.

Progressive infection was defined by two quantitative ELISA p27 antigenaemia results (EDTA whole blood; interval ⩾8 weeks) and a high proviral load on quantitative PCR (qPCR) (>4 × 10^5^ copies/ml).^4,13,15,51^

All cats had at least one FeLV-related condition (anaemia and/or other cytopenias, lymphoma, leukaemia or myelodysplastic syndrome, recurrent infections compatible with immunosuppression or chronic gingivostomatitis)^9,33,52–54^ with poor response to standard therapy, which was maintained throughout raltegravir treatment.

Raltegravir was used as adjunctive therapy. No cat was withdrawn from standard-of-care treatments when clinically indicated, including nutraceuticals or oral feline IFN-ω.^55^ Supportive care followed international guidelines, and adjunctive therapies were maintained throughout the study period.^17,33^

### Ethical approval and informed consent

Written informed consent was obtained from all owners before enrolment. Owners were informed of potential risks and benefits, the voluntary nature of participation and their right to withdraw at any time. The study complied with international standards of veterinary clinical care and was approved by the Ethics and Animal Welfare Commission (CEBEA), Faculty of Veterinary Medicine, Lusófona University (protocol number 120/2022).

### Raltegravir formulation and dosing protocol

Cats received raltegravir (Isentress; MSD Merck Sharp & Dohme), reformulated into 40 or 80 mg gelatin microcapsules by a single independent veterinary compounding pharmacy. Owners purchased capsules in batches of 100; all examinations and tests were funded by the project. The administered dose was 40–80 mg/cat q12h (approximately 10–30 mg/kg PO q12h), based on previous pharmacokinetic and experimental studies.^40,44^ Treatment was generally initiated at 40 mg q12h, with escalation to 80 mg q12h in selected cases during follow-up when considered clinically appropriate.

### Clinical monitoring schedule

Five main clinical–laboratory evaluations were planned: baseline (t0), day 8–10 (t10; safety and tolerance, complete blood count [CBC] and biochemistry),^42,43^ day 45 (t45), day 90 (t90) and day 180 (t180; 90 days after raltegravir discontinuation).

At t0, t45, t90 and t180, each cat underwent an internal medicine consultation with general and FeLV-focused assessment. At these time points, a CBC, proviral DNA load and viral RNA load were obtained. All consultations were performed by the same clinician to minimise inter-observer variability, particularly in assessing FeLV-related manifestations.

### Data collection

Data were retrieved from the clinical management system (Pet Universal) and included age at FeLV diagnosis, date and age at treatment initiation (t0), intrinsic and extrinsic characteristics, clinical assessments, FeLV-related conditions, cause of death, and MST calculated from FeLV diagnosis (MST-D) and from treatment initiation (MST-t0).

For analysis, age was categorised as follows: kitten (birth–6 months), junior (7 months–2 years), adult (3–6 years), mature (7–10 years), senior (11–14 years) and geriatric (>15 years).^56^

### Screening for FeLV p27 antigen and feline immunodeficiency virus antibodies

Point-of-care ELISA (SNAP FIV/FeLV Combo Test; IDEXX Laboratories) was performed on EDTA whole blood, following the manufacturer’s instructions. Testing was performed immediately after sample collection.

### Detection and quantification of FeLV proviral DNA and viral RNA

FeLV proviral DNA (qPCR) and viral RNA (RT-qPCR) were quantified from EDTA whole blood immediately after sample collection. DNA and RNA were extracted using commercial kits (Nzytech) per the manufacturer’s instructions. In line with current guidelines and recent studies, a high proviral load (qPCR >4 × 10^5^ copies/ml) indicates likely progressive infection, whereas 4 × 10^5^ copies/ml or fewer suggests likely regressive infection.^13,15^ All reactions were performed in duplicate using FeLVp dtec-qPCR and FeLV dtec-RT-qPCR kits (Genetic PCR Solutions), manufactured under ISO 9001 and ISO 13485 quality management standards, on a Rotor-Gene Q platform (Qiagen). Standard curves were generated from serial dilutions of the kit-provided template, and appropriate positive and negative controls were included in each run according to the manufacturer’s instructions.

### Laboratory assessments and sample processing

CBCs and serum biochemistry were performed for all cats using a Sysmex XN-1000V analyser (Sysmex) and a Diasys respons 920 analyser (DiaSys Diagnostic Systems), respectively. Haematology included erythrogram, leukogram and thrombogram; biochemistry included alanine aminotransferase, alkaline phosphatase, total bilirubin, albumin and globulin.

CBCs were performed using EDTA blood, whereas biochemistry analyses were performed on heparinised plasma collected at baseline (day 0) and day 10. Results were interpreted according to established feline reference intervals.

Because the cats were naturally infected and exhibited variable disease severity, and because raltegravir was costly, not all 14 cats completed all planned follow-up evaluations. Loss to follow-up was due to clinical deterioration or death, as well as financial or compliance-related limitations.

### Statistical analysis

Statistical analyses were performed using Jamovi software (version 2.5.3; https://www.jamovi.org). Data normality, including viral RNA and proviral DNA loads, was assessed with the Shapiro–Wilk test. Continuous variables are presented as mean ± SD or median, as appropriate, while categorical variables are reported as frequencies and percentages. Proviral DNA and viral RNA loads according to age and treatment time points (t0, t45, t90 and t180) were analysed using the Kruskal–Wallis test, Student’s *t*-test or Wilcoxon signed-rank test, as appropriate. Differences in MST and the effect of different raltegravir doses were evaluated using the Mann–Whitney U-test. Mean survival time was estimated using a single-arm survival analysis. Associations between variables were assessed using Pearson’s or Spearman’s correlation coefficients. Statistical significance was set at *P* <0.05.

## Results

This study included 14 cats with progressive FeLV infection and FeLV‑related diseases/conditions. Three cats died before completing the protocol: cat 7 from toxoplasmosis‑associated sepsis and cats 9 and 10 from leukaemia, at 70, 185 and 78 days, respectively. At the end of the 45‑month observation period, 5/14 cats were still alive. The limited sample size precludes firm epidemiological inferences.

### Study population

Mean age at FeLV diagnosis was 1.92 years (range 7 months to 5.5 years), with most cats ⩽2 years (71.4%), indicating a predominantly young cohort (Table 1). At treatment initiation, mean age was 3.6 years, reflecting a variable interval between diagnosis and antiviral therapy.

**Table 1:** Study population characteristics of 14 FeLV-positive (FELV [+]) cats at treatment initiation (t0)

Cat	Age at FeLV diagnosis (years)	Age at T0 (years)	Sex	Fertile status	Breed	Original background	Lifestyle	Housing conditions	Risk cohabitation	Co-FIV	FeLV complementary treatment	vDNA load (copies/ml)	vRNA load (copies/ml)	FeLV-related conditions
1	0.6	4	Female	Spayed	DSH	Stray/free-roaming cat	Indoor	Multi-cat household	No exposure to FeLV (+) cats	(-)	Nutraceuticals supplements	1.93 × 10^7^	2.40 × 10^7^	Respiratory infections, leukopenia
2	0.7	4	Female	Spayed	DSH	Adopted from shelter	Indoor	Single-cat household	No exposure to FeLV (+) cats	(-)	Interferon feline omega	3.94 × 10^8^	7.66 × 10^9^	FCGE, anaemia
3	1	1	Male	Castrated	DSH	Stray/free-roaming cat	Indoor	Two-cat household	No exposure to FeLV (+) cats	(-)	Interferon feline omega	9.28 × 10^6^	4.50 × 10^9^	FCGE
4	3	4	Female	Spayed	DSH	Stray/free-roaming cat	Indoor	Single-cat household	No exposure to FeLV (+) cats	(-)	Nutraceuticals supplements	3.82 × 10^6^	2.16 × 10^6^	Neutropenia, thrombocytopenia
5	2	4	Female	Spayed	Bengal	Domestic cat	Indoor	Two-cat household	Cohabits with FeLV (+) cat	(-)	N/A	9.93 × 10^5^	1.52 × 10^8^	FCGE
6	2.5	2.5	Male	Castrated	DSH	Stray/free-roaming cat	Outdoor	Single-cat household	Outdoor access	(+)	Interferon feline omega	1.17 × 10^7^	1.78 × 10^9^	FCGE, respiratory infections
7	4.5	5	Male	Castrated	Siamese	Stray/free-roaming cat	Indoor	Single-cat household	No exposure to FeLV (+) cats	(-)	Nutraceuticals supplements	3.34 × 10^6^	2.64 × 10^6^	Anaemia, leukopenia, toxoplasmosis
8	1.5	2	Female	Spayed	DSH	Stray/free-roaming cat	Indoor	Single-cat household	No exposure to FeLV (+) cats	(-)	Interferon feline omega	4.27 × 10^7^	1.70 × 10^7^	FCGE, leukopenia
9	0.6	1.4	Male	Castrated	DSH	Stray/free-roaming cat	Indoor	Single-cat household	No exposure to FeLV (+) cats	(-)	N/A	1.20 × 10^8^	1.39 × 10^7^	Suppurative cholangitis
10	1	3	Male	Castrated	DSH	Stray/free-roaming cat	Outdoor	Single-cat household	Outdoor access	(-)	Nutraceuticals supplements	9.88 × 10^6^	1.53 × 10^8^	Anaemia, thrombocytopenia
11	1.5	2	Female	Spayed	DSH	Stray/free-roaming cat	Indoor	Two-cat household	No exposure to FeLV (+) cats	(-)	N/A	6.57 × 10^5^	1.43 × 10^7^	Anaemia, FCGE
12	5.5	8	Female	Spayed	DSH	Stray/free-roaming cat	Indoor	Multi-cat household	Cohabits with FeLV (+) cat	(-)	N/A	1.75 × 10^6^	4.44 × 10^7^	Lymphoma, anaemia
13	0.5	0.7	Female	Spayed	DSH	Adopted from shelter	Indoor	Single-cat household	No exposure to FeLV (+) cats	(-)	Interferon feline omega	2.47 × 10^6^	2.20 × 10^6^	Respiratory infections
14	2	9	Male	Castrated	DSH	Stray/free-roaming cat	Outdoor	Single-cat household	Outdoor access	(-)	Interferon feline omega	7.87 × 10^5^	4.97 × 10^6^	FCGE, idiopathic ulcerative dermatosis

Co-FIV = co-infection with feline immunodeficiency virus; DSH = domestic shorthair; FCGE = feline chronic gingivostomatitis; N/A = not available; vDNA = proviral DNA load; vRNA = viral RNA load; (+) = positive; (-) = negative

Most cats originated from stray/free-roaming or shelter backgrounds (78.6%) and were client-owned at enrolment. All cats were neutered, predominantly domestic shorthairs and only one cat was co-infected with feline immunodeficiency virus.

### FeLV-related conditions and baseline virological status

At t0, 20 FeLV-related diseases/conditions were recorded in the 14 cats. Chronic gingivostomatitis was most frequent (n = 7/20), followed by persistent or recurrent infections (n = 5/20), anaemia (n = 5/20), persistent leukopenia (n = 4/20), lymphoma (n = 1) and thrombocytopenia (n = 1).

No significant differences in proviral DNA or viral RNA loads were detected between age groups (Kruskal–Wallis, *P* = 0.259 and *P* = 0.927, respectively). At treatment initiation, proviral DNA and viral RNA loads were strongly correlated (*r* = 0.784; *P* <0.001), indicating that higher proviral burden was associated with higher viraemia.

At the 8–10-day follow-up, raltegravir was well tolerated by all cats, with no owner-reported adverse effects or treatment-associated clinicopathological abnormalities detected (Table 2).

**Table 2:** Haematological and biochemical findings at the 8–10-day follow-up after raltegravir treatment initiation

Cat	Erythrogram	Leukogram	Thrombogram	ALT	ALP	Tbil	ALB	GLOB
1	WRI	WRI	WRI	WRI	WRI	N/A	N/A	N/A
2	↓ NN*	↓ LYM	^† ‡^	WRI	WRI	N/A	N/A	N/A
3	WRI	WRI	WRI	WRI	WRI	N/A	N/A	N/A
4	↓ MN*	↓ LYM	*	WRI	N/A	WRI	N/A	N/A
5	WRI	WRI	WRI	WRI	WRI	N/A	N/A	N/A
6	WRI	WRI	WRI	WRI	WRI	WRI	WRI	↑
7	↓ NN*	↓ NEU*	WRI	WRI	N/A	WRI	N/A	N/A
8	WRI	↓ LYM	^† ‡^	WRI	N/A	WRI	N/A	N/A
9	WRI	WRI	WRI	WRI	WRI	N/A	N/A	N/A
10	↓ NN*	↓ LYM	WRI	N/A	N/A	N/A	N/A	N/A
11	↓ MH*	↑ MON	^† ‡^	N/A	N/A	N/A	N/A	N/A
12	↓ NN*	↑ NEU ↑ MON*	WRI	WRI	WRI	N/A	WRI	WRI
13	WRI	WRI	WRI	N/A	N/A	N/A	N/A	N/A
14	WRI	WRI	WRI	N/A	N/A	N/A	N/A	N/A

Leukogram findings indicate decreases (↓) or increases (↑) in neutrophils (NEU) (1.7–8.8 × 10^9^/l), lymphocytes (LYM) (1.2–10.2 × 10^9^/l) or monocytes (MON) (0.1–0.6 × 10^9^/l). Thrombogram findings refer to platelet counts (150–550 × 10^9^/l)

* Pre-existing abnormalities

† Platelet aggregation

‡ Thrombocytopenia

ALB = albumin (31–43 g/l); ALP = alkaline phosphatase (7–68 U/l); ALT = alanine aminotransferase (33–119 U/l); GLOB = globulin (33–42 g/l); MH = macrocytic/hypochromic; MN = macrocytic/normochromic; N/A = not available; NN = normocytic/normochromic; Tbil = total bilirubin (0–6.8 µmol/l); WRI = within reference interval

### Clinical and laboratory monitoring of proviral DNA and viral RNA loads

**Figure 1:**
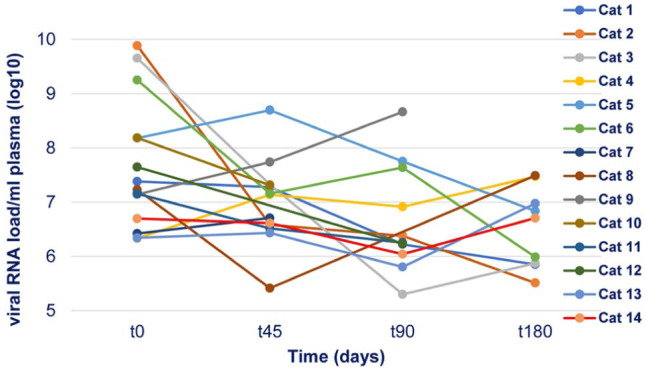
Individual viral RNA loads (log_10_ copies/ml) in 14 progressive feline leukaemia virus-positive cats at t0, t45, t90 and t180, showing the variation in response to raltegravir treatment

**Figure 2:**
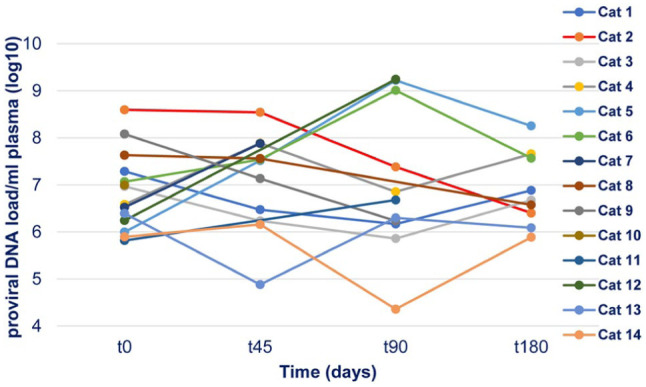
Individual proviral DNA loads (log_10_ copies/ml) in 14 progressive feline leukaemia virus-positive cats at t0, t45, t90 and t180, showing the variation in response to raltegravir treatment

Clinical and laboratory monitoring of proviral DNA and viral RNA loads are shown in Table 3 and Figures 1 and 2.

**Table 3:** Viral RNA load and clinical outcomes of the 14 progressively infected cats during and after raltegravir treatment

Cat	t0	t45		t90		t180		Survival status	Cause of death	MST-D (months)	MST-T0 (days)
	log_10_ vRNA	log_10_ vRNA(ΔT0)	FeLV-related conditions outcomes	log_10_ vRNA(ΔT0)	FeLV-related conditions outcomes	log_10_ vRNA (ΔT0)	FeLV- related diseases outcome				
1	7.380	7.279 (↓0.10)	Improved	6.223 (↓1.05)	Unchanged	5.852 (↓0.37)	Unchanged	Alive	N/A	>60.0	>305.0
2	9.884	6.585 (↓3.30)	Improved	6.378 (↓0.20)	Unchanged	5.512 (↓0.87)	Unchanged	Alive	N/A	60.0	>305.0
3	9.653	7.324 (↓2.33)	Unchanged	5.301 (↓2.02)	Unchanged	5.873 (↑0.57)	Unchanged	Deceased	Lymphoma	24.0	270.0
4	6.334	7.137 (↑0.80)	Improved	6.915 (↓0.22)	Unchanged	7.471 (↑0.56)	Unchanged	Deceased	Leukaemia/myelodysplastic process	24.0	330.0
5	8.182	8.696 (↑0.51)	Unchanged	7.751 (↓0.95)	Unchanged	6.837 (↓0.91)	Unchanged	Alive	N/A	>36.0	>400.0
6	9.250	7.158 (↓2.09)	Improved	7.639 (↑0.45)	Worsened	5.989 (↓1.65)	Unchanged	Alive	N/A	>14.4	>455.0
7*	6.422	6.708 (↑0.29)	Worsened	–	–	–	–	Deceased	Leukaemia/myelodysplastic process	9.6	70.0
8	7.230	5.415 (↓1.82)	Unchanged	–	–	7.489 (—)	Unchanged	Alive	N/A	>42.0	>485.0
9	7.143	7.739 (↑0.60)	Resolved	8.665 (↑0.93)	Resolved	–	–	Deceased	Leukaemia/myelodysplastic process	22.8	185.0
10	8.185	7.312 (↓0.87)	Improved	–	–	–	–	Deceased	Leukaemia/myelodysplastic process	27.6	78.0
11	7.155	6.516 (↓0.64)	Improved	6.255 (↓0.26)	Resolved	–	–	Deceased	Anaemia	48.0	395.0
12	7.647	–	–	6.230 (—)	Worsened	–	–	Deceased	Lymphoma	96.0	210.0
13	6.342	6.433 (↑0.09)	Improved	5.806 (↓0.63)	Improved	6.976 (↑1.17)	Unchanged	Deceased	Leukaemia/myelodysplastic process	25.2	293.0
14	6.696	6.613 (↓0.08)	Improved	6.041 (↓0.57)	Unchanged	6.707 (↑0.67)	Unchanged	Deceased	Lymphoma	102.0	425.0

Viral RNA load and evolution of feline leukemia virus (FeLV)-related conditions at each monitoring point (t0, t45, t90 and t180), including median survival time calculated from diagnosis (MST-D) and median survival time calculated from treatment initiation (MST-T0), and cause of death when applicable. ↓ and ↑ indicate decrease or increase in vRNA load, respectively

* Cat 7 received 80 mg/cat q12h from treatment initiation (t0)

– = not available; ΔT0 = change relative to baseline (t0); N/A = not applicable; vRNA = viral RNA load

### Response during the first 45-day treatment period (t45)

Of the 14 cats, 13 initiated treatment at 40 mg, while one cat started at 80 mg at t0. Viral RNA load was quantified at t45 in 12 cats receiving 40 mg, showing a mean decrease from 1.19 × 10^9^ to 5.46 × 10^7^ copies/ml (21.8-fold; 1.34 log_10_ reduction), which did not reach statistical significance (Wilcoxon signed-rank test, W = 56; *P* = 0.204). At the individual level, viral RNA load increased in 5/13 cats at t45 (Table 3).

### Response during the second 45-day treatment period (t90)

Among the four cats evaluated at day 90 while continuing raltegravir at 40 mg, mean viral RNA load decreased from 1.65 × 10^7^ copies/ml at t0 to 1.31 × 10^6^ copies/ml (≈12.6-fold; 1.10 log_10_), without reaching statistical significance (paired *t*-test, t(3) = 1.60; *P* = 0.208).

In the seven cats receiving 80 mg and assessed at day 90, mean viral RNA load decreased from 1.77 × 10^9^ to 7.25 × 10^7^ copies/ml (≈24.4-fold; 1.39 log_10_), also without statistical significance (Wilcoxon signed-rank test, W = 28; *P* = 0.195).

No dose-dependent effect on viral RNA load reduction was detected between the 40 mg and 80 mg groups (independent *t*-test; *P* = 0.403). At the individual level, viral RNA load increased in 2/11 cats at t90 compared with t45 (Table 3). Two cats died between days 45 and 90, and one additional cat was not evaluated at t90.

### Post-treatment monitoring period (t180)

In the nine cats re-evaluated 90 days after raltegravir discontinuation (t180), no significant changes in viral RNA load were detected (Wilcoxon tests, *P* = 0.641 and *P* = 0.844). Mean viral RNA load (n = 9) decreased from 1.43 × 10^7^ to 6.72 × 10^6^ copies/ml (≈2.1-fold; 0.33 log_10_). At the individual level, viral RNA load increased in 4/9 cats at t180 compared with t90 (Table 3). All cats remained clinically stable, with no new complications or relapses during follow-up, except for one cat that developed acute deterioration and died at day 185 and was therefore not evaluated at t180.

Across the treatment and follow-up period, proviral DNA load showed heterogeneous fluctuations with no consistent treatment-associated trend (Figure 2). Only cats 13 and 14 (14.3%) transitioned from high‑positive to low‑positive proviral DNA load (⩽4 × 10^5^ copies/ml), consistent with a shift from ‘likely progressive’ to ‘likely regressive’ infection.

When the trajectories of proviral DNA and viral RNA loads were analysed at t45, t90 and t180, no significant correlations between the two parameters were identified (*r* = –0.099, *P* = 0.785; *r* = –0.125, *P* = 0.689; *r* = 0.017, *P* = 0.965, respectively). These results indicate heterogeneous viral kinetics, in which higher proviral DNA load was not consistently associated with higher viral RNA levels over time.

### General clinical evolution of FeLV-related conditions during the study period

Across the 180-day monitoring period, most FeLV-related conditions remained clinically stable or showed some degree of improvement (Table 3). Overall, clinical deterioration was observed in only three cats, irrespective of dose or treatment duration. Six cats continued oral feline IFN-ω therapy; no significant differences in proviral DNA or viral RNA loads were detected between interferon-treated and non-treated cats (Mann–Whitney tests, *P* = 0.268 and *P* = 0.149, respectively). All interferon-treated cats remained clinically stable at t180; however, interpretation is limited by the small sample size and data dispersion.

### Survival analysis

Five cats were alive at study closure and were therefore censored for survival analyses. MST was calculated from two reference points: date of FeLV diagnosis (MST-D) and initiation of raltegravir therapy (MST-t0).

**Figure 3:**
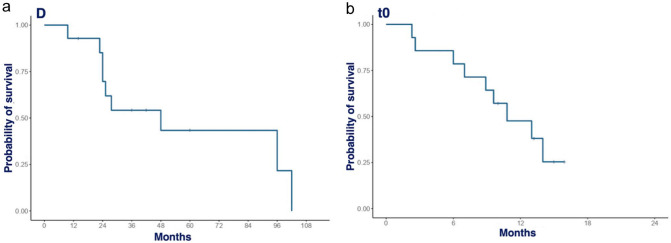
Kaplan–Meier survival analysis of the 14 cats showing survival from (a) the date of feline leukaemia virus diagnosis (D) and (b) initiation of raltegravir treatment (t0)

MST-D (n = 14) was 48 months (1440 days; 95% confidence interval [CI] 24–N/A). Estimated survival probabilities at 12, 36 and 60 months were 93.0%, 54.0% and 43.0%, respectively (Figure 3a). Survival from diagnosis did not differ significantly between age groups (Mann–Whitney U = 10.5; *P* = 0.202), and no correlation was observed between age at diagnosis and MST-D (*r* = 0.194; *P* = 0.507). Interpretation is limited by the small cohort and uneven age distribution at diagnosis (junior 44.8%, adult 28.6%).

From treatment initiation, MST-t0 was 10.8 months (324 days; 95% CI 8.9–N/A). Estimated survival probabilities at 1, 6 and 12 months were 100%, 78.6% and 47.6%, respectively (Figure 3b). No significant differences in MST-t0 were detected between age groups (Kruskal–Wallis χ² = 0.0499; *P* = 0.975), and no correlation was found between age at treatment start and MST-t0 (*r* = –0.024; *P* = 0.935).

Baseline proviral DNA and viral RNA loads were not significantly associated with survival when considering either MST-D (*r* = 0.109, *P* = 0.710; *r* = 0.004, *P* = 0.989, respectively) or MST-t0 (*r* = −0.034, *P* = 0.908; *r* = 0.040, *P* = 0.893). Within this progressive (‘high-positive’) cohort, baseline virological burden was therefore not clearly predictive of survival.

Leukaemia/myelodysplastic syndrome was the most frequent cause of death (n = 5/9, 55.6%), followed by lymphoma (n = 3/9, 33.3%); one cat was euthanased because of treatment-refractory immune-mediated haemolytic anaemia.

## Discussion

Despite decades of research, no specific antiviral therapy has yet been established for the effective treatment of FeLV infection. To the authors’ knowledge, this is the first prospective longitudinal study evaluating the effects of the integrase inhibitor raltegravir in naturally infected, FeLV-progressive cats with FeLV-related conditions, assessing both virological parameters (proviral DNA and viral RNA loads) and clinical outcomes. In total, 14 cats were monitored over 180 days, including 90 days of treatment followed by a 90-day treatment-free period, allowing evaluation of on-treatment and post-treatment effects.

To date, only three studies have assessed raltegravir in FeLV infection: one experimental study in seven cats with induced progressive infection^44^ and two reports in naturally infected cats (two regressive case reports^45^ and one case series of 10 progressive cases).^46^ The latter prospective field study reported clinical monitoring and virological assessment of FeLV RNA load and p27 antigen levels during treatment, although some cats received concurrent antiviral therapies.

At diagnosis, most cats were classified as junior or young adults, mixed-breed and originated from stray or shelter environments. Overall, this study population reflects the typical profile of FeLV-infected cats described in previous studies. Although younger cats are generally considered more susceptible to FeLV infection, epidemiological studies have also reported higher frequencies of infection among adult cats, particularly those with outdoor access or a stray/free-roaming background.^1,3,17,57^

At treatment initiation, proviral DNA and viral RNA loads were strongly correlated (*r* = 0.789; *P* <0.001), supporting an association between higher proviral burden and increased viraemia in progressive FeLV infection. Although based on a limited sample, this finding aligns with previous reports^58,59^ and highlights the potential epidemiological value of proviral load quantification in cats with sustained viral shedding. Longitudinal data assessing both parameters in naturally infected cats remain scarce.

Raltegravir was well tolerated, with no relevant physical or behavioural adverse effects detected at early clinical reassessment, in agreement with previous reports in cats.^44–46^

After 45 days of raltegravir treatment at 40 mg/cat q12h, viral RNA load decreased by a mean of 1.34 log_10_ in cats with paired measurements; this downward trend persisted at day 90, with mean reductions of 1.10 log_10_ in cats maintaining the same dose and 1.39 log_10_ in those receiving 80 mg/cat q12h. These findings are comparable to the only experimental FeLV study, in which an approximately 1 log_10_ reduction was reported after 9 weeks of treatment.^44^ In contrast, the previously reported case series of 10 progressively infected cats did not describe comparable reductions in viral RNA load.^46^ Differences between the studies may reflect variations in study populations, inclusion criteria and virological monitoring protocols. The lack of statistical significance despite these reductions likely reflects the small sample size and the considerable inter-individual variability in viral load responses.

Although reference thresholds for virological response in FeLV infection are lacking, the magnitude of viral RNA reduction observed exceeds the 0.5 log_10_ benchmark commonly used to define antiviral efficacy in human immunodeficiency virus type 1 (HIV-1) infection.^60^ Interpretation remains cautious, as raltegravir is rarely used as monotherapy in HIV-1^61^ or simian immunodeficiency virus (SIV) infection,^62^ and viral loads in those species are typically lower than in FeLV-progressive cats. Nevertheless, the consistent decline observed over 90 days may indicate a potential antiviral effect of raltegravir on FeLV viraemia and raises the question of whether longer treatment duration could further enhance viral suppression. When viral RNA load was reassessed 90 days after treatment discontinuation (t180), no statistically significant changes were detected. Further controlled studies are required to better define the role of raltegravir in the management of progressive FeLV infection.

Regarding raltegravir dosing, no relevant differences in viral RNA load reduction were observed between the 40 mg and 80 mg regimens. This finding is consistent with previous in vitro data indicating that a dose of 40 mg/cat q12h achieves plasma concentrations sufficient to ensure 99% inhibition or higher of FeLV replication.^40^ Moreover, available pharmacokinetic data suggest that increasing the dose beyond 80 mg/cat is unlikely to result in a meaningful increase in plasma exposure or antiviral efficacy.^44^

Raltegravir did not influence proviral DNA load during treatment or follow-up. This is consistent with observations in HIV-1^61^ and SIV infection,^62^ as well as with the only previous in vivo veterinary study in experimentally infected cats.^44^ As an integrase inhibitor, raltegravir prevents new viral integration but does not affect already integrated proviral DNA, which cannot be distinguished by qPCR.^61^ Only one cat shifted from a high- to a low-positive proviral DNA status, supporting the need for multimodal antiviral approaches in progressive FeLV infection.

Regarding FeLV-related conditions, the most frequently diagnosed entities among the 14 cats during the 180-day monitoring period were chronic gingivostomatitis, recurrent or refractory infections – particularly of the respiratory tract – and anaemia, followed by other cytopenias. In most cases, these conditions remained clinically stable or showed some degree of improvement during the treatment and observation period. Leukaemia/myelodysplastic syndrome was the leading cause of death, in 5/9 (55.6%) cats, followed by lymphoma in 3/9 (33.3%) cats; one cat was euthanased because of treatment-refractory immune-mediated haemolytic anaemia. These findings are consistent with previous reports identifying these conditions as major contributors to morbidity and mortality in FeLV-infected cats.^53,54,63,64^ However, in the absence of a control group and given the concurrent use of supportive therapies, no causal relationship between raltegravir treatment and clinical evolution can be inferred.

The median survival time from FeLV diagnosis was 48 months (4 years), with estimated survival probabilities of 93.0%, 54.0% and 43.0% at 12, 36 and 60 months, respectively. Previous studies have reported substantially shorter survival for cats with progressive FeLV infection, often ranging from less than 1 year to 3 years.^8–12^ The longer survival observed here may partly reflect the diagnostic approach at initial presentation, as some cats were first classified as FeLV positive based solely on point-of-care ELISA testing. It is therefore possible that a proportion of cats were initially in a regressive phase, leading to an overestimation of survival when calculated from the first positive test result.

All cats met the inclusion criteria of confirmed progressive FeLV infection and at least one FeLV-related condition, reflecting a real-world clinical population, with several cats severely ill at treatment initiation. Of the 14 cats, three died during the treatment period, which likely influenced the median survival time from treatment start (10.8 months, 324 days), with estimated survival probabilities of 100%, 78.6% and 47.6% at 1, 6 and 12 months, respectively. In contrast, previous studies of raltegravir in FeLV-infected cats primarily included asymptomatic cats^44^ or lacked clear clinical characterisation,^46^ limiting comparability. The potential effect of raltegravir on survival in progressive FeLV infection therefore remains uncertain and warrants evaluation in longer-term studies.

This study has several limitations, including the small sample size and absence of a control group. The effects of raltegravir over a longer treatment duration and its impact on survival could not be assessed, particularly in clinically stable or asymptomatic FeLV-progressive cats. In addition, anti-FeLV antibody responses were not evaluated, limiting insight into host immune modulation during antiviral therapy.

This study aimed to generate real-world data to inform the design of future controlled clinical trials, potentially within multimodal therapeutic strategies. As in HIV-1 infection, where raltegravir is used as a component of highly active antiretroviral therapy,^65^ the integration of raltegravir into combination treatment protocols – if proven effective – could be of substantial value in the management of progressive FeLV infection. Well-designed, randomised, double-blind, placebo-controlled clinical trials in naturally FeLV-infected cats are therefore warranted to robustly evaluate efficacy, safety and the role of antiviral combination therapies in mitigating the clinical consequences of progressive FeLV infection.

Although no highly effective antiviral treatment for FeLV infection is currently available, optimal outcomes rely on a comprehensive management approach, including timely diagnosis, vaccination of at-risk cats, appropriate health monitoring and early identification of comorbidities.^17,33,34^

## Conclusions

Overall, the findings of this study suggest that raltegravir may be associated with reductions in viral RNA load and stabilisation or improvement of FeLV-related clinical manifestations in some cats with progressive FeLV infection, although a clear virological or survival benefit could not be conclusively demonstrated.

These results partially support the study hypothesis but should be interpreted cautiously because of the small sample size and the absence of a control group. Nevertheless, this study provides clinically relevant real-world data that may help inform the design of future long-term, controlled, multimodal antiviral trials targeting different stages of the viral replication cycle in naturally FeLV-infected cats.
